# Synthesis and crystal structure of [2,7,12-trimethyl-3,7,11,17-tetra­aza­bicyclo­[11.3.1]hepta­deca-1(17),13,15-triene-κ^4^ *N*]copper(II) bis­(perchlorate)

**DOI:** 10.1107/S2056989016009701

**Published:** 2016-06-21

**Authors:** Edward Gabrielle V. Hilvano, Guang Yang, Inno A. Emnacen, Elena V. Rybak-Akimova, Junie B. Billones, Maria Constancia O. Carrillo, Bruce C. Noll, Voltaire G. Organo

**Affiliations:** a Department of Physical Sciences and Mathematics, College of Arts and Sciences, University of the Philippines, Manila 1000, Philippines; b Department of Chemistry, Tufts University, Medford, Massachusetts 02155, USA; c Bruker AXS Inc., 5465 E. Cheryl Parkway, Madison, WI 53711, USA

**Keywords:** crystal structure, pyridine macrocycles, copper complex

## Abstract

The title copper(II) complex of a pyridine-containing macrocycle (PyMAC), [Cu(C_16_H_28_N_4_)](ClO_4_)_2_, has been prepared. The crystal structure reveals the Cu^II^ atom to be octahedrally coordinated by a tetradentate aminopyridine macrocyclic ligand surrounding the metal cation in a square-planar geometry. Two weakly bound perchlorate counter-ions occupy the axial sites above and below the macrocyclic plane. The crystal studied was refined as a two-component pseudo-merohedral twin; the refined fractional contribution of the minor component is 38.77 (8)%

## Abstract 2

A copper(II) complex of a pyridine-containing macrocycle (PyMAC) reveals a six-coordinated octa­hedral Cu^II^ complex with a tetra­dentate amino­pyridine macrocycle ligand surrounding the metal centre in a square-planar geometry. Two weakly bound perchlorate counter-ions occupy the axial sites above and below the macrocyclic plane.

## Chemical context   

**Figure 1:**
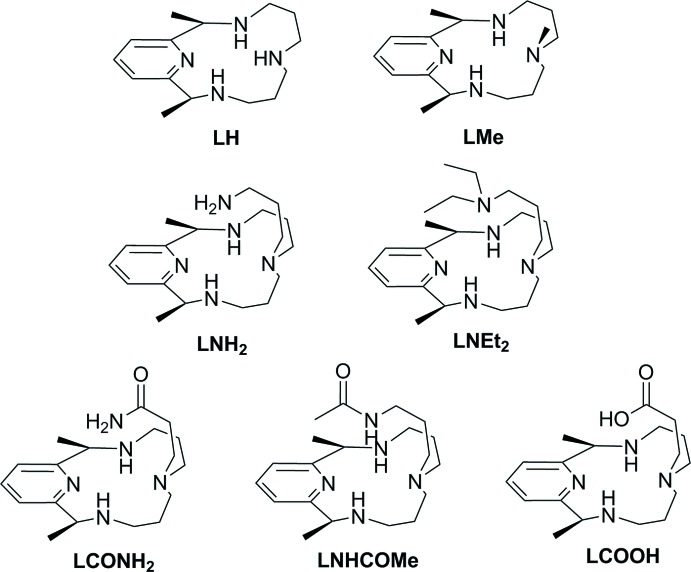
Pyridine-containing macrocycles (PyMACs).

There have been several studies of the macrocycles synthesized from 2,6-di­acetyl­pyridine and polyamines. One of the first examples, reported by Karn & Busch (1966 ▸), involved a nickel(II)-templated condensation of 2,6-di­acetyl­pyridine and bis­(3-amino­prop­yl)amine. Their pioneering work enabled subsequent syntheses of various pyridine-containing macrocycles (Rezaeivala & Keypour, 2014 ▸), including a family of complexes with appended arms (PyMACs) (Organo *et al.*, 2009 ▸; Herrera *et al.*, 2003 ▸) as shown in Fig. 1 ▸.

Various metal ions have been incorporated into PyMAC ligands, and the resulting complexes often showed inter­esting catalytic properties. For example, Ni^II^–PyMAC complexes have been found to exhibit peroxidase-like activity, with NiLCOOH (Fig. 1 ▸) being most active (Organo *et al.*, 2009 ▸). Fe^II^–LMe (Fig. 1 ▸) was also found to have catalytic use in epoxidation reactions of cyclo­octene with hydrogen peroxide (Ye *et al.*, 2012 ▸). A similar Cu^II^–PyMAC complex but without methyl groups at the macrocyclic ring was reported by Fernandes *et al.* (2007 ▸) to scavenge superoxide.

Pyridine-containing metallomacrocycles have also found utility beyond synthetic chemistry. For example, Cu–macrocyclic complexes have become increasingly important in radiopharmaceutical applications as contrast agents in positron emission tomographic (PET) imaging (Boros *et al.*, 2014 ▸).

While there are known Cu–pyridine macrocycles, only a few have been characterized structurally (Caira *et al.*, 1975 ▸; Lindoy *et al.*, 2001 ▸; Herrera *et al.*, 2003 ▸; Autzen *et al.*, 2003 ▸). Here, we report the synthesis and crystal structure of a Cu^II^–PyMAC perchlorate compound.
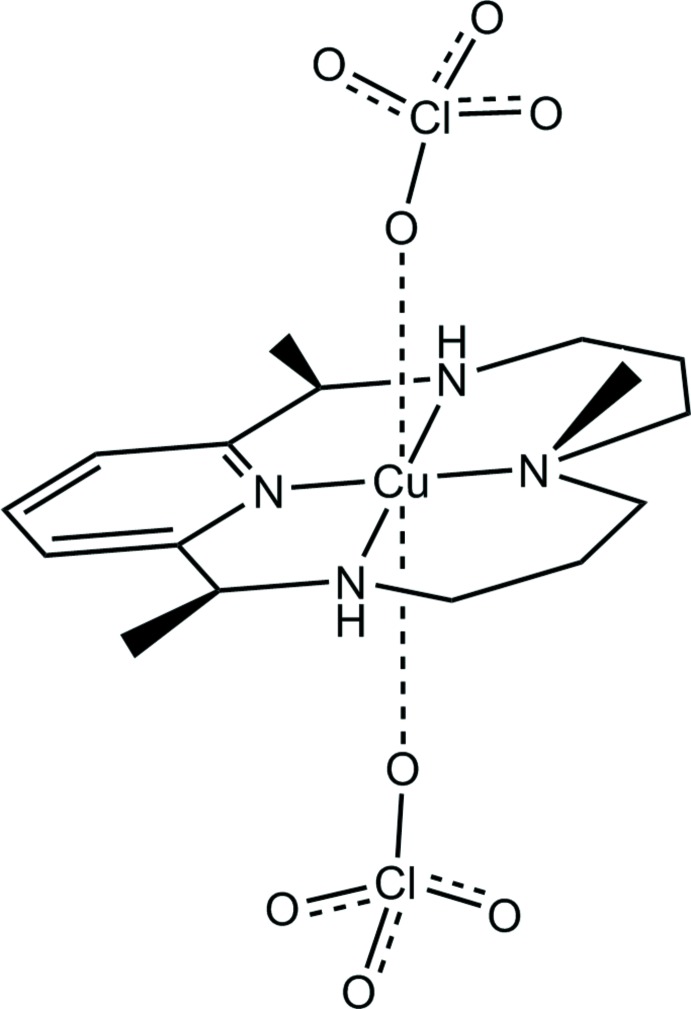


## Structural commentary   

**Figure 2:**
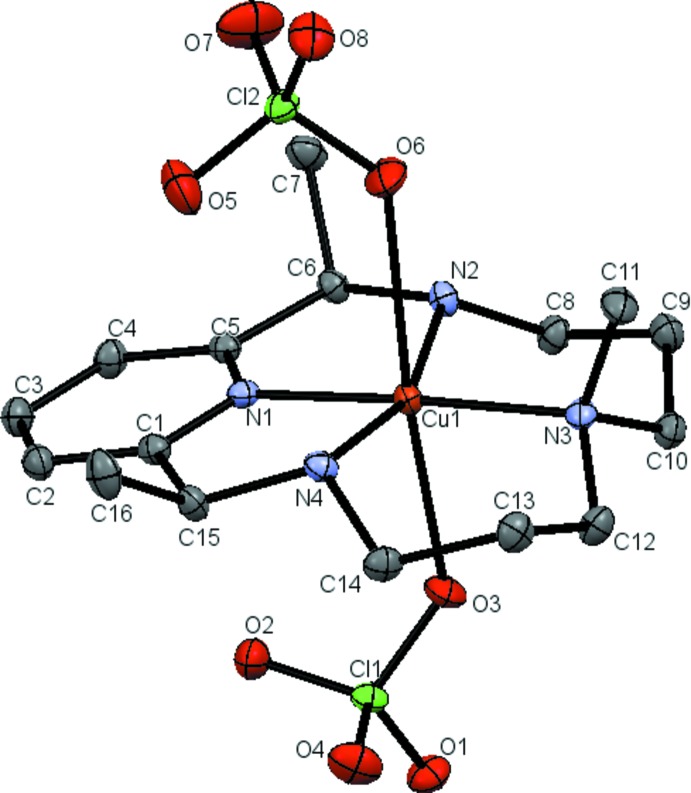
An *ORTEP* diagram of the mol­ecular structure of Cu*L*Me(ClO_4_)_2_ [*L*Me = 2,7,12-trimethyl-3,7,11,17-tetra­aza­bicyclo­[11.3.1]hepta­deca-1(17),13,15-triene, see Fig. 1 ▸], showing the atom-labeling scheme, with ellipsoids drawn at the 50% probability level. Hydrogen atoms are omitted for clarity.

The title compound has the Cu^II^ atom in a distorted octahedral coordination, with the tetradentate amino­pyridine macro­cyclic ligand surrounding the metal atom in a square-planar geometry (Fig. 2 ▸). Two perchlorate counter-ions occupy the axial sites perpendicular to the macrocyclic plane. The macrocyclic ligand incorporates a 2,6-substituted pyridine unit that is connected on both sides to an aliphatic chain of 11 atoms, including two secondary amines and a tertiary amine bearing a methyl group. When coordinated to the Cu^II^ atom, the macrocycle exhibits approximate mol­ecular mirror symmetry with respect to the plane that bis­ects the pyridine and tertiary amine nitro­gen atoms, and is perpendicular to the macrocyclic plane. The Cu—N distances between Cu^II^ and secondary amine nitro­gen atoms [2.0417 (14) and 2.0445 (15) Å] are similar to each other; the distance between Cu^II^ and the tertiary amine N atom [2.0108 (13) Å] is slightly shorter. In contrast, the Cu—N_py_ bond length [1.9316 (13) Å] is much shorter than the Cu—N_amine_ bonds. Both perchlorate anions are only weakly bound, with Cu—O6 and Cu—O3 distances of 2.6478 (13) and 2.4736 (13) Å, respectively.

An intra­molecular contact (N4—H4⋯O5) occurs between a perchlorate O atom and the tertiary amine NH group. The N⋯O distance [3.423 (2) Å] is longer than the sum of van der Waals radii of the two atoms (2.94 Å), suggesting this is a weaker inter­action comparing to normal hydrogen-bonding interactions.

## Supra­molecular features   

**Figure 3:**
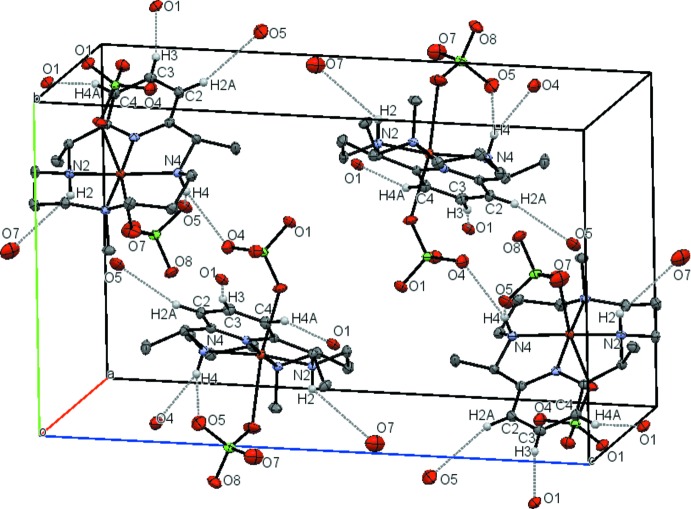
Crystal packing of the title complex viewed approximately down the *a* axis. Hydrogen atoms, except those involved in hydrogen bonds, are omitted for clarity.

In the crystal of the complex (see Fig. 3 ▸), several N—H⋯O and C_py_—H⋯O hydrogen bonds have longer *D*⋯*A* distances than the van der Waals radii of the corresponding pairs of atoms (3.25 Å for C⋯O). The resulting geometry is a chain along [010]. Numerical details are given in Table 1 ▸.

## Synthesis and crystallization   

The procedure for the synthesis of the title compound was adapted from Karn & Busch (1966 ▸) with subsequent reduction using NaBH_4_. 10 mmol of 2,6-di­acetyl­pyridine were dissolved in 160 ml of absolute ethanol, and the resulting solution was mixed with 10 mmol of Cu(ClO_4_)_2_·6H_2_O in 240 ml of water. The reaction mixture was heated to 338 K and 10 mmol of *N*,*N*-bis­(3-amino­prop­yl)methyl­amine were added. Subsequently, glacial acetic acid was added to the mixture until the pH was about 4. The mixture was heated to reflux of the solvent for 12 h; a color change from blue to dark blue occurred during that period. After reflux, the mixture was cooled to room temperature and 40 mmol of NaBH_4_ were added. The mixture was left to stir for 12 h for complete reduction. Perchloric acid was added until the remaining NaBH_4_ was consumed.

The deep-blue solution was concentrated to about a tenth of its original volume by rotary evaporation. The solution was then cooled slowly to room temperature and refrigerated. Dark-purple needle-like crystals formed upon cooling. The crystals were filtered, washed with absolute ethanol and diethyl ether, and allowed to dry. Light-purple crystals were recrystallized from hot water. Single crystals were obtained by dissolving the compound in aceto­nitrile followed by slow ether diffusion.

UV–Vis data: λ_max_ = 552 nm in methanol, molar extinction coefficient: 209.47 *M* ^−1^·cm^−1^. IR: 1619 cm^−1^ (C=N of pyridine), 1113 and 600 cm^−1^ (ClO_4_^−^ bands) and 3400 cm^−1^ (N—H).

## Refinement   

Crystal data, data collection and structure refinement details are summarized in Table 2 ▸. The crystal structure was refined as a two-component pseudo-merohedral twin (twin operation: 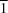
00, 0
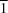
0, 001); the refined fractional contribution of the minor component is 38.77 (8)%. All H atoms bonded to C atoms were placed at calculated positions using a riding model, with C—H distances of 0.98 Å for CH, 0.97 Å for CH_2_, 0.96 Å for CH_3_, and 0.93 Å for aromatic CH, and with *U*_iso_(H) = 1.2*U*_eq_(C) for all but CH_3_ where *U*_iso_(H) = 1.5*U*_eq_(C). H2 and H4 connected to N2 and N4 were located in the difference density Fourier synthesis maps and refined freely.

## Supplementary Material

Crystal structure: contains datablock(s) I. DOI: 10.1107/S2056989016009701/zl2666sup1.cif

Structure factors: contains datablock(s) I. DOI: 10.1107/S2056989016009701/zl2666Isup2.hkl

Supporting information file. DOI: 10.1107/S2056989016009701/zl2666sup3.tif

CCDC reference: 1485717

Additional supporting information: crystallographic information; 3D view; checkCIF report

## Figures and Tables

**Table 1:** Hydrogen-bond geometry (Å, °)

*D*—H⋯*A*	*D*—H	H⋯*A*	*D*⋯*A*	*D*—H⋯*A*
N2—H2⋯O7^i^	0.83 (2)	2.94 (2)	3.536 (2)	130.6 (18)
N4—H4⋯O4^ii^	0.86 (2)	2.45 (2)	3.1619 (19)	140.2 (18)
N4—H4⋯O5	0.86 (2)	2.77 (2)	3.423 (2)	134.1 (17)
C2—H2*A*⋯O5^iii^	0.93	2.70	3.587 (2)	161
C3—H3⋯O1^iv^	0.93	2.68	3.585 (2)	165
C4—H4*A*⋯O1^v^	0.93	2.64	3.518 (2)	158

Symmetry codes: (i) 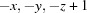; (ii) 
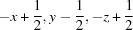
; (iii) 
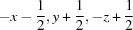
; (iv) 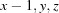; (v) 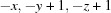.

**Table 2:** Experimental details

Crystal data	
Chemical formula	[Cu(C_16_H_28_N_4_)](ClO_4_)_2_
*M*_r_	538.86
Crystal system, space group	Monoclinic, *P*2_1_/*n*
Temperature (K)	100
*a*, *b*, *c* (Å)	8.6918 (12), 12.0588 (16), 20.068 (3)
β (°)	90.153 (3)
*V* (Å^3^)	2103.4 (5)
*Z*	4
Radiation type	Mo *K*α
μ (mm^−1^)	1.35
Crystal size (mm)	0.24 × 0.21 × 0.21

Data collection	
Diffractometer	Bruker D8 QUEST
Absorption correction	Multi-scan (Krause *et al.*, 2015 ▸)
*T*_min_, *T*_max_	0.414, 0.454
No. of measured, independent and observed [*I* > 2σ(*I*)] reflections	36478, 5284, 5054
*R*_int_	0.030
(sin θ/λ)_max_ (Å^−1^)	0.687

Refinement	
*R*[*F* ^2^ > 2σ(*F* ^2^)], *wR*(*F* ^2^), *S*	0.026, 0.062, 1.06
No. of reflections	5284
No. of parameters	292
H-atom treatment	H atoms treated by a mixture of independent and constrained refinement
Δρ_max_, Δρ_min_ (e Å^−3^)	0.50, −0.33

Computer programs: *APEX2* and *SAINT* (Bruker, 2013 ▸), *SHELXT* (Sheldrick, 2015*a* ▸), *SHELXL2014* (Sheldrick, 2015*b* ▸), *Mercury* (Macrae *et al.*, 2006 ▸), *OLEX2* (Dolomanov *et al.*, 2009 ▸) and *publCIF* (Westrip, 2010 ▸).

## supplementary crystallographic information

### Crystal data

**Table:**

[Cu(C_16_H_28_N_4_)](ClO_4_)_2_	*F*(000) = 1116
*M**_r_* = 538.86	*D*_x_ = 1.702 Mg m^−^^3^
Monoclinic, *P*2_1_/*n*	Mo *K*α radiation, λ = 0.71073 Å
*a* = 8.6918 (12) Å	Cell parameters from 9732 reflections
*b* = 12.0588 (16) Å	θ = 2.9–30.6°
*c* = 20.068 (3) Å	µ = 1.35 mm^−^^1^
β = 90.153 (3)°	*T* = 100 K
*V* = 2103.4 (5) Å^3^	Clear dark blue cube, clear dark blue
*Z* = 4	0.24 × 0.21 × 0.21 mm

### Data collection

**Table:**

Bruker D8 QUEST diffractometer	5284 independent reflections
Radiation source: sealed tube	5054 reflections with *I* > 2σ(*I*)
Detector resolution: 1.024 pixels mm^-1^	*R*_int_ = 0.030
φ and ω scans	θ_max_ = 29.2°, θ_min_ = 2.9°
Absorption correction: multi-scan (Krause *et al.*, 2015)	*h* = −11→11
*T*_min_ = 0.414, *T*_max_ = 0.454	*k* = −16→16
36478 measured reflections	*l* = −27→27

### Refinement

**Table:**

Refinement on *F*^2^	0 restraints
Least-squares matrix: full	Hydrogen site location: mixed
*R*[*F*^2^ > 2σ(*F*^2^)] = 0.026	H atoms treated by a mixture of independent and constrained refinement
*wR*(*F*^2^) = 0.062	*w* = 1/[σ^2^(*F*_o_^2^) + (0.027*P*)^2^ + 0.369*P*] where *P* = (*F*_o_^2^ + 2*F*_c_^2^)/3
*S* = 1.06	(Δ/σ)_max_ = 0.002
5284 reflections	Δρ_max_ = 0.50 e Å^−^^3^
292 parameters	Δρ_min_ = −0.33 e Å^−^^3^

### Special details

**Table:**

Geometry. All esds (except the esd in the dihedral angle between two l.s. planes) are estimated using the full covariance matrix. The cell esds are taken into account individually in the estimation of esds in distances, angles and torsion angles; correlations between esds in cell parameters are only used when they are defined by crystal symmetry. An approximate (isotropic) treatment of cell esds is used for estimating esds involving l.s. planes.
Refinement. Refined as a 2-component twin.

### Fractional atomic coordinates and isotropic or equivalent isotropic displacement parameters (Å^2^)

**Table:**

	*x*	*y*	*z*	*U*_iso_*/*U*_eq_	
Cu1	0.19755 (3)	0.24103 (2)	0.38162 (2)	0.01124 (5)	
N1	0.00263 (15)	0.31793 (10)	0.38014 (7)	0.0121 (2)	
N2	0.14981 (17)	0.22614 (12)	0.48078 (7)	0.0131 (3)	
H2	0.131 (3)	0.1590 (18)	0.4820 (11)	0.016 (5)*	
N3	0.40687 (15)	0.17015 (10)	0.38456 (7)	0.0133 (2)	
N4	0.16917 (17)	0.24951 (11)	0.28059 (7)	0.0133 (3)	
H4	0.126 (2)	0.1870 (18)	0.2721 (10)	0.014 (5)*	
C1	−0.05040 (19)	0.35796 (13)	0.32233 (8)	0.0132 (3)	
C2	−0.1787 (2)	0.42658 (14)	0.32010 (8)	0.0158 (3)	
H2A	−0.2162	0.4538	0.2799	0.019*	
C3	−0.24897 (18)	0.45302 (14)	0.37997 (9)	0.0169 (3)	
H3	−0.3339	0.4999	0.3803	0.020*	
C4	−0.1932 (2)	0.40991 (13)	0.43957 (8)	0.0160 (3)	
H4A	−0.2407	0.4268	0.4798	0.019*	
C5	−0.06452 (19)	0.34082 (13)	0.43793 (8)	0.0126 (3)	
C6	0.00692 (19)	0.28859 (13)	0.49932 (8)	0.0135 (3)	
H6	0.0363	0.3483	0.5299	0.016*	
C7	−0.1071 (2)	0.21384 (15)	0.53495 (9)	0.0191 (3)	
H7A	−0.1304	0.1510	0.5073	0.029*	
H7B	−0.1998	0.2544	0.5438	0.029*	
H7C	−0.0630	0.1888	0.5762	0.029*	
C8	0.2787 (2)	0.24911 (14)	0.52748 (9)	0.0162 (4)	
H8A	0.2451	0.2360	0.5728	0.019*	
H8B	0.3086	0.3264	0.5238	0.019*	
C9	0.4161 (2)	0.17602 (15)	0.51239 (8)	0.0186 (3)	
H9A	0.3824	0.0993	0.5121	0.022*	
H9B	0.4906	0.1842	0.5481	0.022*	
C10	0.4954 (2)	0.20050 (14)	0.44663 (8)	0.0159 (3)	
H10A	0.5183	0.2792	0.4450	0.019*	
H10B	0.5927	0.1611	0.4460	0.019*	
C11	0.3963 (2)	0.04734 (13)	0.38084 (10)	0.0193 (3)	
H11A	0.3423	0.0199	0.4191	0.029*	
H11B	0.4979	0.0162	0.3799	0.029*	
H11C	0.3418	0.0265	0.3411	0.029*	
C12	0.5038 (2)	0.21068 (15)	0.32814 (9)	0.0174 (3)	
H12A	0.6037	0.1750	0.3311	0.021*	
H12B	0.5200	0.2897	0.3337	0.021*	
C13	0.4389 (2)	0.19068 (15)	0.25850 (9)	0.0177 (3)	
H13A	0.5213	0.1989	0.2264	0.021*	
H13B	0.4023	0.1148	0.2558	0.021*	
C14	0.3084 (2)	0.26795 (13)	0.23923 (8)	0.0164 (3)	
H14A	0.3422	0.3441	0.2444	0.020*	
H14B	0.2825	0.2564	0.1927	0.020*	
C15	0.04536 (19)	0.33025 (14)	0.26215 (8)	0.0142 (3)	
H15	0.0967	0.3989	0.2484	0.017*	
C16	−0.0511 (2)	0.29131 (16)	0.20309 (8)	0.0216 (4)	
H16A	0.0151	0.2758	0.1660	0.032*	
H16B	−0.1229	0.3483	0.1909	0.032*	
H16C	−0.1061	0.2253	0.2151	0.032*	
Cl1	0.31018 (5)	0.52884 (3)	0.37452 (2)	0.01419 (7)	
O1	0.40486 (16)	0.60559 (10)	0.41159 (6)	0.0200 (2)	
O2	0.15073 (13)	0.55835 (10)	0.38070 (7)	0.0214 (2)	
O3	0.33372 (15)	0.41828 (10)	0.40105 (7)	0.0221 (3)	
O4	0.35382 (17)	0.53023 (11)	0.30525 (6)	0.0254 (3)	
Cl2	−0.09242 (5)	0.01135 (3)	0.35753 (2)	0.01541 (8)	
O5	−0.14975 (19)	0.09033 (13)	0.30953 (8)	0.0323 (4)	
O6	0.05744 (15)	0.04607 (11)	0.38012 (8)	0.0289 (3)	
O7	−0.1936 (2)	0.00640 (14)	0.41356 (8)	0.0373 (4)	
O8	−0.08306 (17)	−0.09626 (11)	0.32766 (7)	0.0249 (3)	

### Atomic displacement parameters (Å^2^)

**Table:**

	*U*^11^	*U*^22^	*U*^33^	*U*^12^	*U*^13^	*U*^23^
Cu1	0.01167 (8)	0.01105 (8)	0.01101 (8)	0.00167 (6)	−0.00066 (9)	0.00045 (6)
N1	0.0128 (6)	0.0102 (5)	0.0133 (6)	−0.0014 (4)	0.0012 (5)	0.0002 (5)
N2	0.0142 (6)	0.0122 (6)	0.0130 (6)	0.0025 (5)	−0.0011 (5)	−0.0005 (5)
N3	0.0126 (6)	0.0100 (5)	0.0173 (6)	−0.0007 (5)	−0.0024 (5)	−0.0011 (5)
N4	0.0152 (8)	0.0105 (6)	0.0144 (6)	0.0008 (5)	0.0011 (5)	0.0002 (4)
C1	0.0128 (7)	0.0127 (7)	0.0141 (7)	−0.0009 (5)	−0.0001 (6)	−0.0001 (6)
C2	0.0154 (8)	0.0162 (7)	0.0156 (7)	0.0016 (6)	−0.0027 (6)	0.0016 (6)
C3	0.0140 (6)	0.0167 (7)	0.0201 (7)	0.0034 (5)	0.0009 (6)	0.0001 (7)
C4	0.0144 (7)	0.0185 (7)	0.0151 (7)	0.0023 (6)	0.0018 (6)	−0.0011 (6)
C5	0.0136 (7)	0.0123 (7)	0.0121 (7)	−0.0014 (6)	−0.0006 (5)	0.0005 (5)
C6	0.0151 (8)	0.0129 (7)	0.0124 (7)	0.0017 (6)	0.0004 (6)	0.0001 (6)
C7	0.0206 (8)	0.0182 (8)	0.0185 (8)	0.0021 (7)	0.0052 (7)	0.0038 (6)
C8	0.0149 (10)	0.0211 (8)	0.0126 (7)	−0.0006 (6)	−0.0029 (6)	0.0000 (6)
C9	0.0173 (8)	0.0214 (8)	0.0170 (8)	0.0015 (7)	−0.0028 (6)	0.0033 (6)
C10	0.0136 (7)	0.0173 (8)	0.0167 (7)	0.0000 (6)	−0.0031 (6)	0.0001 (6)
C11	0.0196 (7)	0.0119 (7)	0.0264 (8)	0.0012 (6)	−0.0035 (7)	−0.0011 (7)
C12	0.0131 (8)	0.0208 (8)	0.0182 (8)	−0.0005 (6)	0.0011 (6)	−0.0015 (6)
C13	0.0167 (8)	0.0188 (8)	0.0176 (8)	0.0026 (6)	0.0034 (6)	−0.0024 (6)
C14	0.0171 (8)	0.0175 (7)	0.0147 (7)	−0.0001 (7)	0.0037 (7)	−0.0007 (5)
C15	0.0162 (8)	0.0142 (7)	0.0122 (7)	0.0033 (6)	0.0014 (6)	0.0020 (5)
C16	0.0247 (9)	0.0277 (9)	0.0124 (7)	0.0078 (7)	−0.0030 (6)	0.0000 (7)
Cl1	0.01690 (16)	0.01039 (15)	0.01528 (15)	−0.00062 (12)	0.00114 (15)	−0.00024 (12)
O1	0.0203 (6)	0.0171 (6)	0.0225 (6)	−0.0045 (5)	−0.0008 (5)	−0.0051 (5)
O2	0.0157 (5)	0.0206 (6)	0.0280 (6)	0.0019 (4)	−0.0006 (5)	0.0012 (5)
O3	0.0252 (7)	0.0109 (5)	0.0303 (6)	−0.0007 (5)	−0.0027 (5)	0.0048 (5)
O4	0.0359 (8)	0.0230 (7)	0.0175 (6)	0.0010 (6)	0.0083 (5)	−0.0005 (5)
Cl2	0.01338 (16)	0.01372 (16)	0.01913 (17)	−0.00071 (13)	0.00050 (15)	−0.00212 (13)
O5	0.0370 (9)	0.0249 (7)	0.0349 (8)	0.0108 (6)	−0.0117 (6)	0.0023 (6)
O6	0.0200 (6)	0.0197 (6)	0.0470 (8)	−0.0074 (5)	−0.0096 (7)	0.0012 (6)
O7	0.0401 (9)	0.0351 (8)	0.0368 (8)	−0.0028 (7)	0.0230 (8)	−0.0050 (6)
O8	0.0283 (7)	0.0176 (6)	0.0287 (7)	0.0024 (5)	−0.0031 (6)	−0.0091 (5)

### Geometric parameters (Å, º)

**Table:**

Cu1—N1	1.9316 (13)	C8—H8B	0.9700
Cu1—N2	2.0417 (14)	C8—C9	1.515 (3)
Cu1—N3	2.0108 (13)	C9—H9A	0.9700
Cu1—N4	2.0444 (15)	C9—H9B	0.9700
Cu1—O3	2.4736 (13)	C9—C10	1.519 (2)
Cu1—O6	2.6478 (13)	C10—H10A	0.9700
N1—C1	1.337 (2)	C10—H10B	0.9700
N1—C5	1.329 (2)	C11—H11A	0.9600
N2—H2	0.83 (2)	C11—H11B	0.9600
N2—C6	1.500 (2)	C11—H11C	0.9600
N2—C8	1.485 (2)	C12—H12A	0.9700
N3—C10	1.507 (2)	C12—H12B	0.9700
N3—C11	1.4857 (19)	C12—C13	1.525 (2)
N3—C12	1.496 (2)	C13—H13A	0.9700
N4—H4	0.86 (2)	C13—H13B	0.9700
N4—C14	1.486 (2)	C13—C14	1.517 (3)
N4—C15	1.497 (2)	C14—H14A	0.9700
C1—C2	1.389 (2)	C14—H14B	0.9700
C1—C15	1.506 (2)	C15—H15	0.9800
C2—H2A	0.9300	C15—C16	1.524 (2)
C2—C3	1.386 (2)	C16—H16A	0.9600
C3—H3	0.9300	C16—H16B	0.9600
C3—C4	1.390 (2)	C16—H16C	0.9600
C4—H4A	0.9300	Cl1—O1	1.4436 (13)
C4—C5	1.395 (2)	Cl1—O2	1.4365 (12)
C5—C6	1.515 (2)	Cl1—O3	1.4498 (12)
C6—H6	0.9800	Cl1—O4	1.4421 (13)
C6—C7	1.520 (2)	Cl2—O5	1.4423 (15)
C7—H7A	0.9600	Cl2—O6	1.4403 (13)
C7—H7B	0.9600	Cl2—O7	1.4308 (15)
C7—H7C	0.9600	Cl2—O8	1.4317 (13)
C8—H8A	0.9700		
			
N1—Cu1—N2	82.92 (6)	H8A—C8—H8B	108.0
N1—Cu1—N3	176.39 (5)	C9—C8—H8A	109.4
N1—Cu1—N4	81.79 (6)	C9—C8—H8B	109.4
N1—Cu1—O3	90.40 (5)	C8—C9—H9A	108.6
N1—Cu1—O6	91.30 (5)	C8—C9—H9B	108.6
N2—Cu1—N4	161.21 (6)	C8—C9—C10	114.84 (14)
N2—Cu1—O3	91.20 (5)	H9A—C9—H9B	107.5
N2—Cu1—O6	80.69 (6)	C10—C9—H9A	108.6
N3—Cu1—N2	96.91 (6)	C10—C9—H9B	108.6
N3—Cu1—N4	99.04 (6)	N3—C10—C9	116.03 (14)
N3—Cu1—O3	86.00 (5)	N3—C10—H10A	108.3
N3—Cu1—O6	92.23 (5)	N3—C10—H10B	108.3
N4—Cu1—O3	99.76 (5)	C9—C10—H10A	108.3
N4—Cu1—O6	88.79 (5)	C9—C10—H10B	108.3
O3—Cu1—O6	171.44 (5)	H10A—C10—H10B	107.4
C1—N1—Cu1	119.14 (11)	N3—C11—H11A	109.5
C5—N1—Cu1	118.27 (11)	N3—C11—H11B	109.5
C5—N1—C1	122.07 (14)	N3—C11—H11C	109.5
Cu1—N2—H2	99.0 (15)	H11A—C11—H11B	109.5
C6—N2—Cu1	111.60 (10)	H11A—C11—H11C	109.5
C6—N2—H2	108.8 (16)	H11B—C11—H11C	109.5
C8—N2—Cu1	116.33 (11)	N3—C12—H12A	108.3
C8—N2—H2	108.1 (15)	N3—C12—H12B	108.3
C8—N2—C6	111.95 (13)	N3—C12—C13	115.72 (14)
C10—N3—Cu1	112.38 (10)	H12A—C12—H12B	107.4
C11—N3—Cu1	111.48 (10)	C13—C12—H12A	108.3
C11—N3—C10	108.37 (13)	C13—C12—H12B	108.3
C11—N3—C12	108.83 (13)	C12—C13—H13A	108.7
C12—N3—Cu1	110.52 (10)	C12—C13—H13B	108.7
C12—N3—C10	105.00 (12)	H13A—C13—H13B	107.6
Cu1—N4—H4	101.8 (14)	C14—C13—C12	114.27 (14)
C14—N4—Cu1	117.72 (11)	C14—C13—H13A	108.7
C14—N4—H4	112.3 (14)	C14—C13—H13B	108.7
C14—N4—C15	110.53 (13)	N4—C14—C13	112.03 (13)
C15—N4—Cu1	111.25 (10)	N4—C14—H14A	109.2
C15—N4—H4	101.8 (14)	N4—C14—H14B	109.2
N1—C1—C2	121.18 (15)	C13—C14—H14A	109.2
N1—C1—C15	115.19 (14)	C13—C14—H14B	109.2
C2—C1—C15	123.48 (14)	H14A—C14—H14B	107.9
C1—C2—H2A	121.2	N4—C15—C1	110.16 (13)
C3—C2—C1	117.68 (15)	N4—C15—H15	106.9
C3—C2—H2A	121.2	N4—C15—C16	112.66 (14)
C2—C3—H3	119.8	C1—C15—H15	106.9
C2—C3—C4	120.40 (15)	C1—C15—C16	112.84 (14)
C4—C3—H3	119.8	C16—C15—H15	106.9
C3—C4—H4A	120.6	C15—C16—H16A	109.5
C3—C4—C5	118.75 (15)	C15—C16—H16B	109.5
C5—C4—H4A	120.6	C15—C16—H16C	109.5
N1—C5—C4	119.91 (14)	H16A—C16—H16B	109.5
N1—C5—C6	116.31 (14)	H16A—C16—H16C	109.5
C4—C5—C6	123.77 (15)	H16B—C16—H16C	109.5
N2—C6—C5	110.17 (13)	O1—Cl1—O3	108.70 (8)
N2—C6—H6	108.1	O2—Cl1—O1	110.21 (8)
N2—C6—C7	111.11 (13)	O2—Cl1—O3	109.36 (8)
C5—C6—H6	108.1	O2—Cl1—O4	109.67 (9)
C5—C6—C7	111.28 (14)	O4—Cl1—O1	109.77 (8)
C7—C6—H6	108.1	O4—Cl1—O3	109.11 (8)
C6—C7—H7A	109.5	Cl1—O3—Cu1	132.04 (8)
C6—C7—H7B	109.5	O6—Cl2—O5	109.19 (9)
C6—C7—H7C	109.5	O7—Cl2—O5	109.90 (10)
H7A—C7—H7B	109.5	O7—Cl2—O6	108.79 (11)
H7A—C7—H7C	109.5	O7—Cl2—O8	109.08 (9)
H7B—C7—H7C	109.5	O8—Cl2—O5	109.81 (9)
N2—C8—H8A	109.4	O8—Cl2—O6	110.05 (9)
N2—C8—H8B	109.4	Cl2—O6—Cu1	132.63 (8)
N2—C8—C9	111.04 (14)		
			
Cu1—N1—C1—C2	−171.12 (12)	C3—C4—C5—N1	0.4 (2)
Cu1—N1—C1—C15	4.49 (19)	C3—C4—C5—C6	−179.88 (15)
Cu1—N1—C5—C4	170.68 (12)	C4—C5—C6—N2	−175.87 (15)
Cu1—N1—C5—C6	−9.09 (18)	C4—C5—C6—C7	60.4 (2)
Cu1—N2—C6—C5	2.44 (16)	C5—N1—C1—C2	0.5 (2)
Cu1—N2—C6—C7	126.24 (12)	C5—N1—C1—C15	176.06 (14)
Cu1—N2—C8—C9	−56.21 (16)	C6—N2—C8—C9	173.83 (13)
Cu1—N3—C10—C9	55.43 (16)	C8—N2—C6—C5	134.81 (14)
Cu1—N3—C12—C13	−58.20 (16)	C8—N2—C6—C7	−101.40 (16)
Cu1—N4—C14—C13	48.28 (16)	C8—C9—C10—N3	−70.04 (19)
Cu1—N4—C15—C1	−15.39 (16)	C10—N3—C12—C13	−179.61 (15)
Cu1—N4—C15—C16	−142.35 (12)	C11—N3—C10—C9	−68.21 (18)
N1—C1—C2—C3	0.6 (2)	C11—N3—C12—C13	64.54 (18)
N1—C1—C15—N4	7.8 (2)	C12—N3—C10—C9	175.62 (15)
N1—C1—C15—C16	134.62 (15)	C12—C13—C14—N4	−66.11 (19)
N1—C5—C6—N2	3.89 (19)	C14—N4—C15—C1	−148.11 (14)
N1—C5—C6—C7	−119.80 (16)	C14—N4—C15—C16	84.93 (17)
N2—C8—C9—C10	67.86 (19)	C15—N4—C14—C13	177.62 (13)
N3—C12—C13—C14	75.39 (19)	C15—C1—C2—C3	−174.60 (15)
C1—N1—C5—C4	−1.0 (2)	O1—Cl1—O3—Cu1	169.81 (9)
C1—N1—C5—C6	179.27 (14)	O2—Cl1—O3—Cu1	49.44 (13)
C1—C2—C3—C4	−1.2 (2)	O4—Cl1—O3—Cu1	−70.51 (12)
C2—C1—C15—N4	−176.75 (15)	O5—Cl2—O6—Cu1	22.21 (15)
C2—C1—C15—C16	−49.9 (2)	O7—Cl2—O6—Cu1	−97.73 (14)
C2—C3—C4—C5	0.7 (3)	O8—Cl2—O6—Cu1	142.81 (11)

### Hydrogen-bond geometry (Å, º)

**Table:**

*D*—H···*A*	*D*—H	H···*A*	*D*···*A*	*D*—H···*A*
N2—H2···O7^i^	0.83 (2)	2.94 (2)	3.536 (2)	130.6 (18)
N4—H4···O4^ii^	0.86 (2)	2.45 (2)	3.1619 (19)	140.2 (18)
N4—H4···O5	0.86 (2)	2.77 (2)	3.423 (2)	134.1 (17)
C2—H2*A*···O5^iii^	0.93	2.70	3.587 (2)	161
C3—H3···O1^iv^	0.93	2.68	3.585 (2)	165
C4—H4*A*···O1^v^	0.93	2.64	3.518 (2)	158

Symmetry codes: (i) −*x*, −*y*, −*z*+1; (ii) −*x*+1/2, *y*−1/2, −*z*+1/2; (iii) −*x*−1/2, *y*+1/2, −*z*+1/2; (iv) *x*−1, *y*, *z*; (v) −*x*, −*y*+1, −*z*+1.
