# Supplementary figures and images for: Synthesis and crystal structure of [2,7,12-trimethyl-3,7,11,17-tetra­aza­bicyclo­[11.3.1]hepta­deca-1(17),13,15-triene-κ4 N]copper(II) bis­(perchlorate)
Source: Acta Crystallogr E Crystallogr Commun. 2016 Jun 21;72(Pt 7):1009–12. doi: 10.1107/S2056989016009701 (PMC4992927; doi:10.1107/S2056989016009701)

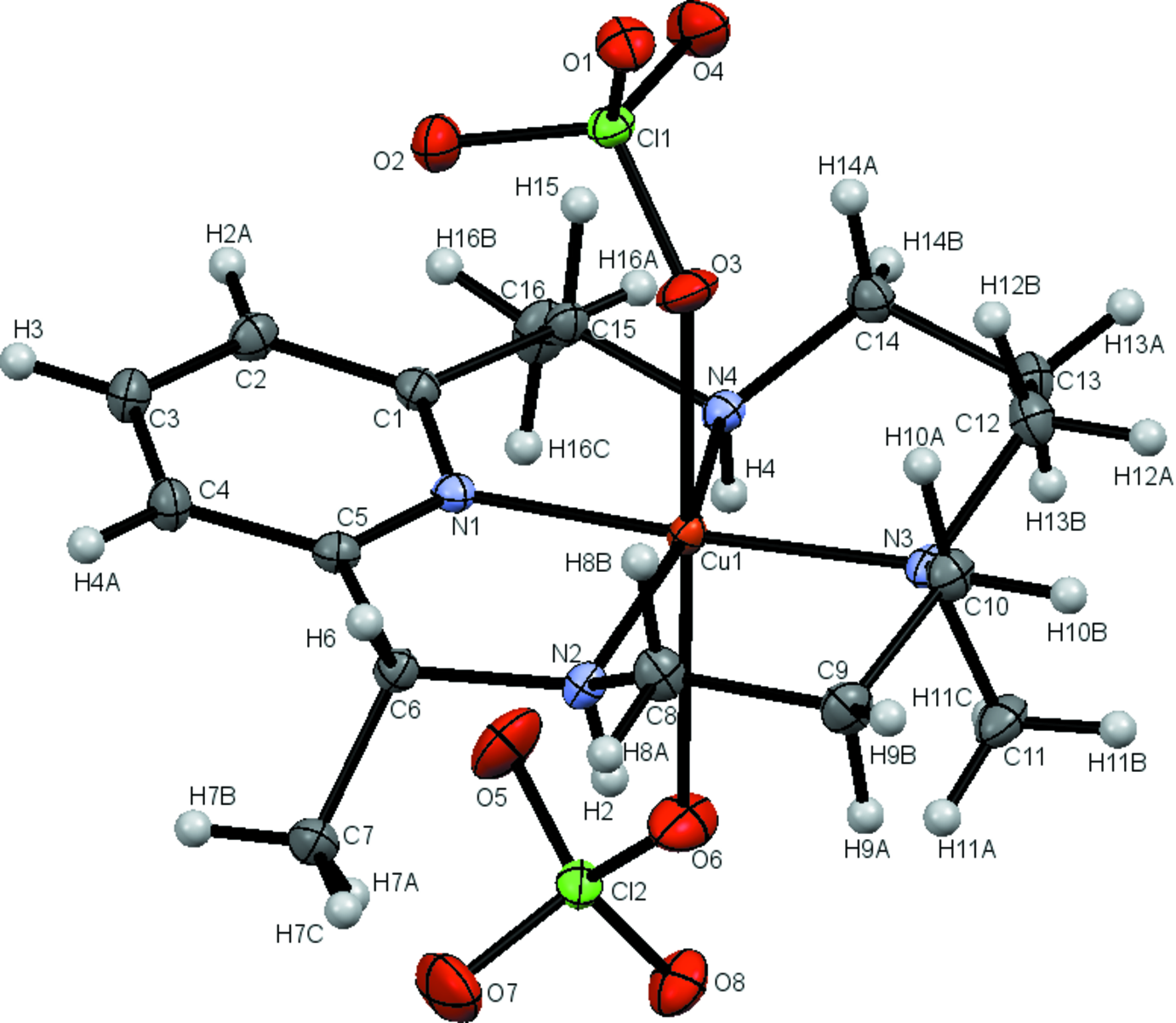

Supplement: Supplementary file 3 [file e-72-01009-sup3.tif]
